# Supplementary material for: Causal Relationship between Aging and Anorexia Nervosa: A White-Matter-Microstructure-Mediated Mendelian Randomization Analysis
Source: Biomedicines. 2024 Aug 16;12(8):1874. doi: 10.3390/biomedicines12081874 (PMC11351342; doi:10.3390/biomedicines12081874)
Supplement: Supplementary file 1 [file biomedicines-12-01874-s001.zip › Supplementary Figure/Figure S4/Singe SNP plot of MR analysis for TL to ubm-b-1838.pdf]

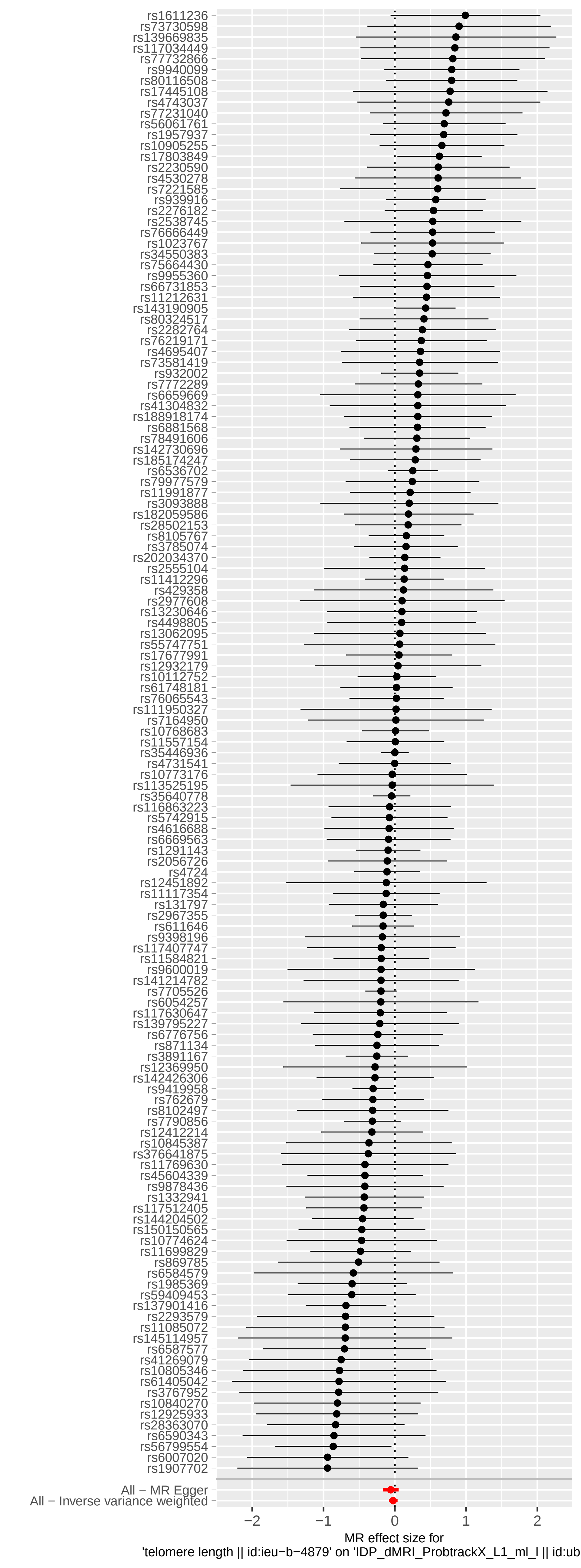
rs1611236
rs73730598
rs139669835
rs117034449
rs77732866
rs9940099
rs80116508
rs17445108
rs4743037
rs77231040
rs56061761
rs1957937
rs10905255
rs17803849
rs2230590
rs4530278
rs7221585
rs939916
rs2276182
rs2538745
rs76666449
rs1023767
rs34550383
rs75664430
rs9955360
rs66731853
rs11212631
rs143190905
rs80324517
rs2282764
rs76219171
rs4695407
rs73581419
rs932002
rs7772289
rs6659669
rs41304832
rs188918174
rs6881568
rs78491606
rs142730696
rs185174247
rs6536702
rs79977579
rs11991877
rs3093888
rs182059586
rs28502153
rs8105767
rs3785074
rs202034370
rs2555104
rs11412296
rs429358
rs2977608
rs13230646
rs4498805
rs13062095
rs55747751
rs17677991
rs12932179
rs10112752
rs61748181
rs76065543
rs111950327
rs7164950
rs10768683
rs11557154
rs35446936
rs4731541
rs10773176
rs113525195
rs35640778
rs116863223
rs5742915
rs4616688
rs6669563
rs1291143
rs2056726
rs4724
rs12451892
rs11117354
rs131797
rs2967355
rs611646
rs9398196
rs117407747
rs11584821
rs9600019
rs141214782
rs7705526
rs6054257
rs117630647
rs139795227
rs6776756
rs871134
rs3891167
rs12369950
rs142426306
rs9419958
rs762679
rs8102497
rs7790856
rs12412214
rs10845387
rs376641875
rs11769630
rs45604339
rs9878436
rs1332941
rs117512405
rs144204502
rs150150565
rs10774624
rs11699829
rs869785
rs6584579
rs1985369
rs59409453
rs137901416
rs2293579
rs11085072
rs145114957
rs6587577
rs41269079
rs10805346
rs61405042
rs3767952
rs10840270
rs12925933
rs28363070
rs6590343
rs56799554
rs6007020
rs1907702
All − MR Egger
All − Inverse variance weighted
−2
−1
0
1
2
MR effect size for
'telomere length || id:ieu−b−4879' on 'IDP_dMRI_ProbtrackX_L1_ml_l || id:ub
